# Study on Prediction Model of HIV Incidence Based on GRU Neural Network Optimized by MHPSO

**DOI:** 10.1109/ACCESS.2020.2979859

**Published:** 2020-03-10

**Authors:** Xiaoming Li, Xianghui Xu, Jie Wang, Jing Li, Sheng Qin, Juxiang Yuan

**Affiliations:** School of Public HealthNorth China University of Science and Technology 128790 Tangshan 063210 China; Hebei Province Key Laboratory of Occupational Health and Safety for Coal IndustryNorth China University of Science and Technology 128790 Tangshan 063210 China; Department of Internal MedicineNorth China University of Science and Technology Hospital Tangshan 063210 China

**Keywords:** AIDS, deep learning, incidence prediction, LSTM network, MHPSO-GRU network, RNN

## Abstract

Acquired Immune Deficiency Syndrome (AIDS) is still one of the most life-threatening diseases in the world. Moreover, new infections are still potentially increasing. This difficult problem must be solved. Early warning is the most effective way to solve this problem. Here, we aim to determine the best performing model to track the epidemic of AIDS, which will provide a methodological basis for testing the time characteristics of the disease. From January 2004 to January 2018, we built four computing methods based on AIDS dataset: BPNN model, RNN model, LSTM model and MHPSO-GRU model. Compare the final estimated performance to determine the preferred method. Result. Considering the root mean square error (RMSE), mean absolute error (MAE), mean error rate (MER) and mean absolute percentage error (MAPE) in the simulation and prediction subsets, the MHPSO-GRU model is determined as the best performance technology. Estimates for the period from May 2018 to December 2020 suggest that the event appears to continue to increase and remain high.

## Graphical abstract

This paper uses RNN derived network GRU to predict the incidence rate of AIDS. The mhpso-gru network prediction model of HIV incidence sequence was established. Mhpso Gru network optimizes the initial weight of deep Gru network through mhpso, and then restores the optimal solution to the initial weight of deep Gru network.

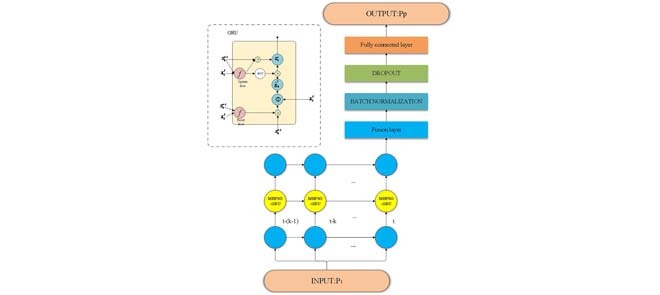

## I. Introduction

Acquired immune deficiency syndrome (AIDS) is the result of human immunodeficiency virus (HIV) infection, and its main feature is T cell immune function defect [1]. HIV mainly exists in the blood, semen, vaginal secretions and milk of infected people. Therefore, it is usually transmitted between people through direct sexual contact, iatrogenic and perinatal transmission [2], [3]. Since 1997, the annual incidence of AIDS in the world has declined slightly, but it is still one of the most important causes of death in the world’s major disease burden [4]–[7]. It is estimated that there were 1.8 million new infections in 2016, with an average life expectancy of 40.32 years. About 70% of these new infections occur in low and middle-income countries, with investment spending reaching 8.13 billion US dollars [8]. In recent years, the number of AIDS cases reported annually has increased [9], [10]. In addition, the mortality rate of AIDS in class B statutory infectious diseases is the highest from 2008 to 2017, and the vaccine has not been determined [11], [12]. Therefore, facing the major public health challenges and threats brought by AIDS, and providing a clear and quantitative direction for effective formulation of preventive plans and rational allocation of limited available resources, this is a high-precision and accurate understanding of the prediction model of AIDS epidemic behavior is necessary. Jia J established an HIV / AIDS epidemic model with general nonlinear incidence and treatment methods, and studied the stability of disease-free balance and unique local balance [13]. Wang Yawen established the combination of autoregressive moving average model, generalized regression neural network and ARIMA model. ARIMA (1,1,1) (0,1,1) 12 and ARIMA-GRNN model can better predict the monthly incidence of AIDS in China, but the effect of the combined model is better [14]. Yang K used nonparametric local smoothing method to develop a flexible and effective data modeling method for St disease incidence, monitoring the incidence of cancer, AIDS, cardiovascular disease and other chronic or infectious diseases, with good results [15]. Mar Masi áused the multi state method to predict the development of PLWH into non AIDS events, and estimated three transfer probabilities: from alive and NAE free to alive and NAE experienced (“NAE development”); from alive and NAE experienced to death (“death after NAE”) [16]. Rezaei s elucidates the overall prevalence of depression in HIV / AIDS patients through systematic review and meta-analysis [17]. Adamu incidence rate in various states affected by insurgency activities in Boko Haram, Nigeria, collected data from 2004 to 2017, reporting 16 [18]. The information of 102 patients, including age, gender, diagnosis year and patient status, found that most of the HIV / AIDS infected people were women in these Boko Haram areas. Although these laws were discovered, they did not give a trend of future incidence rate. Deribew A studied the development trend of HIV / AIDS mortality in Ethiopia from 1990 to 2016, found that the decline of HIV / AIDS mortality was relatively slow, and provided the corresponding HIV testing and treatment plan, but did not give a future development trend, and the plan had a certain lag [19].

Deep learning is a kind of machine learning method to represent data washing. It combines the bottom features of data into the top features through deep neural network, and is used for classification, prediction and other tasks. Compared with the traditional shallow learning methods such as neural network, logic regression, support vector machine, decision tree, etc., its network depth is deeper, its nonlinear representation ability is stronger, and its network learning ability is stronger. Shallow learning is often ineffective in dealing with complex problems, even when the number of samples is sufficient. This is because the network complexity is low and the learning ability is not strong. Even if a large number of samples are input, some important details cannot be learned. However, in the past, due to the limitation of computing power, blindly increasing the number of layers and parameters of the network is easy to lead to slow convergence and over fitting of the network. Today’s deep learning technology can effectively solve these problems. While increasing the network depth, greedy layer by layer training method is used to prevent over fitting and improve the network performance.

## II. RNN and its Derived Neural Network

From the first Turing test to the present deep learning, in recent decades, imitating human brain has always been the direction of artificial intelligence. However, in the past few years, due to the limitation of computing power, computers have been unable to process the same amount of data as human brain. With the rapid development of computer computing power, deep learning model solves this problem through various methods [20]. ANN (artificial neural network) has also developed rapidly and been widely used, and derived convolutional neural network (CNN) with spatial distribution data and recurrent neural network (RNN, recurrent neural network) with temporal distribution data [21]. At the same time, the cyclic neural network model is widely used in time series data processing because it can process the time-series data.

### A. Cyclic Neural Network Model (RNN)

In terms of time series data processing, taking the prediction of AIDS incidence to be discussed in this paper as an example, it is different from the traditional artificial neural network (ANN) that takes a certain time cross-section factor as the input value of data. The RNN takes a certain long-term factor data as the time series and takes the data in the past period as the input value [22].

**FIGURE 1.**
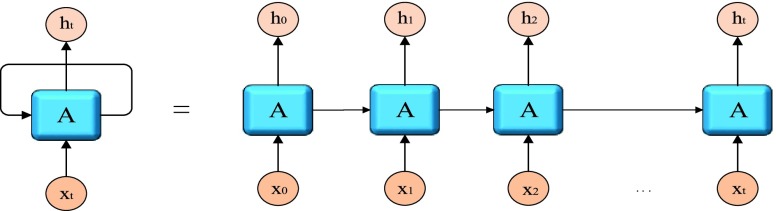
RNN cycle expansion.

Figure 1 is to expand unified neuron $A$ according to time $\left [{ {0,t} }\right]$. the characteristics of chain structure reveal that RNN is essentially related to sequence and list. In this process, we can see the operation of the whole neuron at different time points. The input variable $x_{0} $ is processed in the neuron at the time of 0 and input $h_{0} $. This process is not over, the whole process has entered into 1 moment.

However, due to the high complexity of RNN model, gradient explosion and gradient disappearance are easy to occur when the input variables enter the hidden layer for data processing. In 1994, yoshua bengio explained the gradient disappearance by mathematical formula (1) [23].

\begin{equation*} h_{t} =\delta \left ({{Ux_{t} +Wh_{t-1} +b} }\right)\tag{1}\end{equation*}

Because the hidden layer gradient will multiply the coefficient w repeatedly in the process of back propagation along the time dimension, if the data is long series data, the more times the coefficient W is multiplied repeatedly, the more likely it is to cause the gradient explosion and gradient disappearance.

### B. Long and Short Term Memory Network Model (LSTM)

**FIGURE 2.**
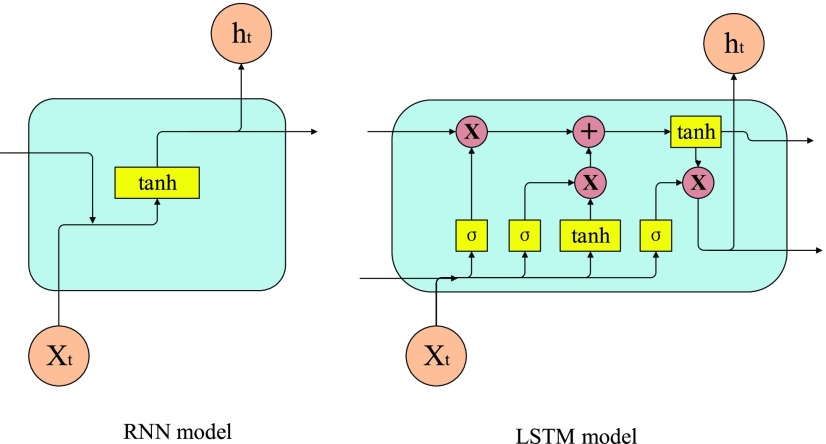
Internal schematic diagram of RNN model and LSTM model.

The solution to RNN problem comes from the introduction of long-term memory network (LSTM) in 1997. Hochrater and schmiduber, the proponents of long-term and short-term memory networks, found that the essential reason for RNN to produce gradient explosion and gradient disappearance is that the calculation method of hidden layer state causes the gradient to be expressed in the form of continuous product, and put forward an improved way to solve this problem [24], as shown in Figure 2.

If we omit $o_{t},~y_{t} $ and $L_{t} $ for each layer, then the model can be simplified as shown in the figure above. In RNN model, we can see clearly that the hidden state $h_{t} $ is determined by $h_{t-1} $ and $x_{t} $ together. On the way through the activation function $\sigma $, which is tanh in general, the LSTM model on the right side of the figure above is much more complex.

RNN neural network in the process of transforming the growing neural network, because of repeated cycles in the hidden layer, the error will become more significant along with the cycle, resulting in the disappearance of gradient, the change of training results is not obvious, and finally the whole training process cannot escape from the local optimal solution [25], [26].

## III. GRU Neural Network

**FIGURE 3.**
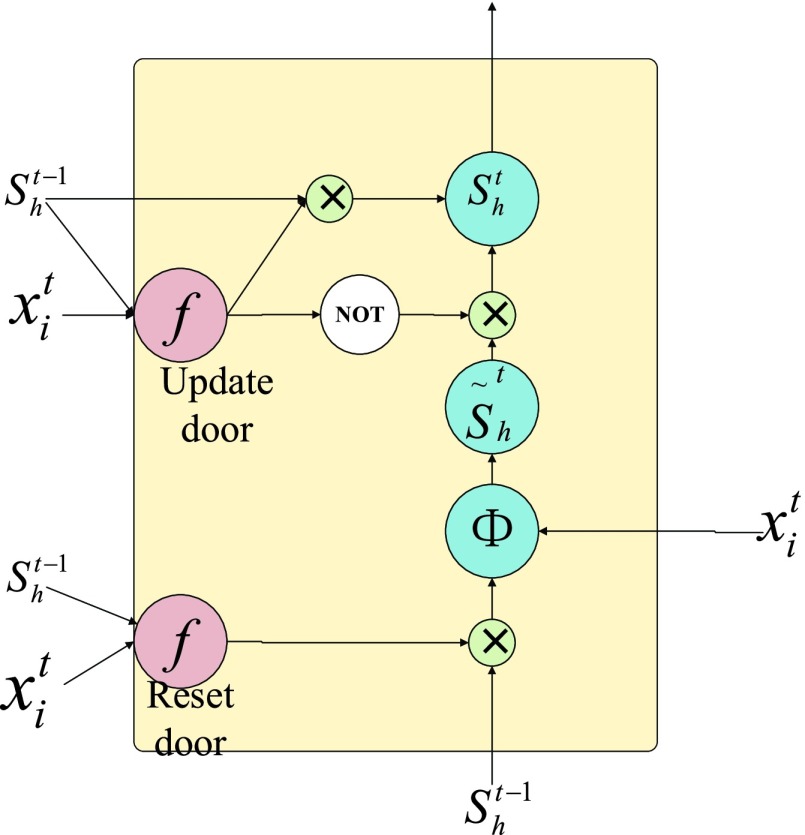
GRU unit structure diagram.

GRU network was proposed in 2014, which is essentially a cyclic neural network and an improved version based on LSTM network [27]. Based on the LSTM unit, the input gate and forgetting gate in LSTM are combined into a single update gate, and the specific structure is shown in Figure 3.

\begin{align*} a_{u}^{t}=&\sum \limits _{i=1}^{I} {w_{iu} x_{i}^{t}} +\sum \limits _{h=1}^{H} {w_{hu} s_{h}^{t-1}} \tag{2}\\ s_{u}^{t}=&f\left ({{a_{u}^{t}} }\right)\tag{3}\\ a_{r}^{l}=&\sum \limits _{i=1}^{I} {w_{ir} x_{i}^{l}} +\sum \limits _{h}^{H} {w_{hr} s_{h}^{t-1}}\tag{4}\\ s_{r}^{t}=&f\left ({{a_{r}^{t}} }\right)\tag{5}\\ \mathop {a_{h'}}\limits ^{\sim ^{t}}=&\sum \limits _{i=1}^{I} {w_{ih'} x_{i}^{t}} +s_{r}^{t} \sum \limits _{h}^{H} {w_{hh'} s_{h}^{t-1}}\tag{6}\end{align*}

**FIGURE 4.**
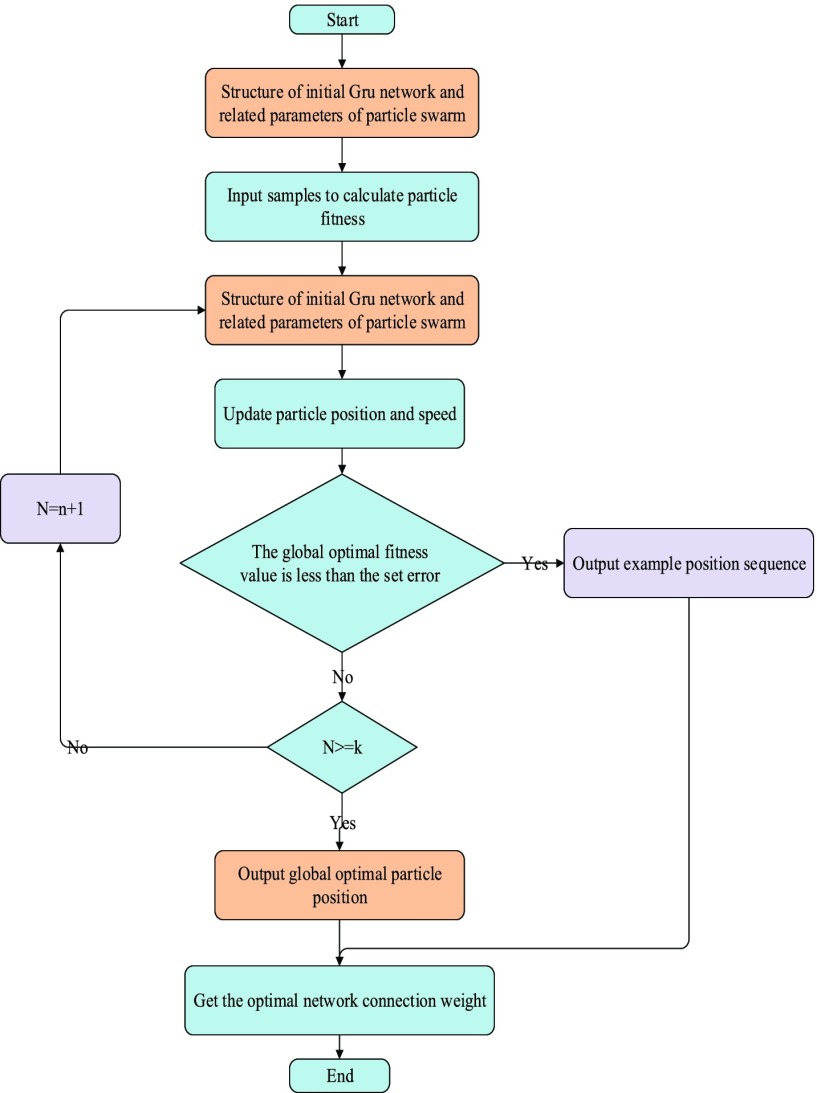
Flow chart of MHPSO-GRU model.

According to Figure 3, the forward propagation formula can be obtained as shown in formula (2)–(6).Where $u_{\thinspace }$ is the index of the updated gate vector, $r_{\thinspace }$ is the index of the reset gate vector, $h_{\thinspace }$ is the index of the hidden cell vector at all times, $h'$ is the index of the new memory cell at $t$, and $f_{\thinspace }$ and $\varphi _{\thinspace }$ are activation functions. $f$ is the sigmoid function, $\varphi _{\thinspace }$ is the tanh function, and $\mathop {s_{h'}}\limits ^{\sim ^{t}}$ represents the information of the new memory unit at time $t$ [28]. From Figure 4, it can be seen that the new memory information is obtained from the synthesis of the hidden unit states at $t_{\thinspace }$ time input: $_{\mathrm {\thinspace }}x_{i}^{t} $ and $t-1$ time, indicating that the new memory can have the new input information and historical information at the same time. The reset gate determines the weight of the hidden unit information in time $t-1$ to the new memory information $\mathop {s_{h'}}\limits ^{\sim t} $ in time [29]. If the current time state is independent of the previous time, the reset door can prevent the transmission of the previous time information. However, there is an update gate as the final output control gate, and the information of the previous moment can also be transmitted through the update gate. The weights of the new memory information and the hidden unit information at time: $t-1$ are respectively controlled by the update gate. The above is the basic working principle of GRU, which can not only solve the problem of RNN gradient disappearing, but also reduce the network parameters compared with LSTM, and has faster convergence speed. Moreover, experiments show that the network performance is even better than LSTM.

### A. GRU Neural Network Based on MHPSO Optimization

Particle swarm optimization (PSO) is a kind of swarm intelligence optimization algorithm, which is proposed by Kennedy and Eberhart in 1995 to simulate the foraging of birds. According to formula (7) and (8), the particles of the population constantly adjust their speed and position to optimize until the convergence termination condition is met.

\begin{align*} V_{i,j}^{t+1}=&\omega V_{i,j}^{t} +c_{1} r_{1} \left ({{y_{i,j}^{t} -x_{i,j}^{t}} }\right)+c_{2} r_{2} \left ({{y_{i}^{t} -x_{i,j}^{t}} }\right)\qquad \quad \tag{7}\\ X_{i,j}^{t+1}=&X_{i,j}^{t} +V_{i,j}^{t+1}\tag{8}\end{align*}

In the formula, $V_{i,j}^{t} $ represents the velocity of the particle $i$ in the j-th dimension at the t-th iteration; $x_{i,j}^{t} $ is the position of the particle $i$; $\omega $ is the inertia weight; $c_{1}$ and $c_{2} $ are acceleration coefficients or learning factors; $y_{i,j}^{t} $ is the t Individual extreme points during the iteration; $y_{i}^{t} $ is the global extreme point of the particle swarm; $r_{1}$, $r_{2} $ are random numbers uniformly distributed in the interval [0, 1]; $V_{i,j}^{t} \in \left [{ {-V_{\max },V_{\max }} }\right]$, $V_{\max } $ are constants.

Aiming at the problem that particle swarm algorithm is easy to fall into the local optimal position, this paper proposes a hierarchical heterogeneous dynamic particle swarm optimization algorithm. The main purpose of this algorithm is to establish a direct connection between the particle swarm structure layer and the layer. The number of particles is the same.

Each time the algorithm iterates, it arranges all particles according to the current fitness value and assigns them to different levels. The adaptive value of the particles is inversely proportional to the level they are in. When the algorithm runs, the particles will Attracted by the particles on the upper layer, these particles are called attracted particles, and the particles themselves will also act as attracted particles of a certain particle in the lower layer. Particles, and it is the attracting particles of the fourth layer of particles; for the particles at the highest and lowest layers, its attracting particles are other particles in the same layer as it. In the process of optimization, the particles will not only The local best and global best positions move, while also moving in the direction determined by its attracting particles.

The MHPSO algorithm incorporates a parameter related to attracting particles in the iterative update formula. The specific display can be seen from the following equation:

\begin{align*}&\hspace {-0.5pc}V_{i,j}^{t+1} =\omega V_{i,j}^{t} +c_{1} r_{1} \left ({{y_{i}^{t} -x_{i,j}^{t}} }\right)+c_{2} r_{2} \left ({{y_{i}^{t} -x_{i,j}^{t}} }\right) \\& \qquad\qquad\qquad\qquad\; \displaystyle {+\sum \nolimits _{a=1}^{A_{j}^{i}} {c_{3} r_{3} \left ({{x\left ({i }\right)_{a,j}^{t} -x_{i,j}^{t}} }\right)}}\tag{9}\end{align*}

Among them, $x\left ({i }\right)_{a,j}^{t} $ represents the position of the attracting particle $a$ of the particle $i$, and B represents the attracting particle $a$ of the particle $i$, $c_{3} $ represents the constant of the acceleration coefficient, and $r_{3} $ represents the attraction coefficient of particle $i$ to attract particle $a$.

The position update formula is shown in equation (10):

\begin{equation*} X_{i,j}^{t+1} =X_{i,j}^{t} +V_{i,j}^{t+1}\tag{10}\end{equation*}

The gradient descent method adopted by GRU neural network is easy to make the weight converge to a certain value, but it can not guarantee that it is the global minimum value of the error plane. The optimization of GRU neural network by MHPSO is to replace the gradient descent method in GRU neural network by GRU. The main idea of this algorithm is to optimize the connection weight and threshold of GRU neural network by MHPSO algorithm, which can improve the GRU neural network Prediction performance.

The specific steps of short-term traffic flow prediction based on optimized GRU neural network parameters of MHPSO are as follows:

(1) build training data set and test data set, and preprocess traffic data to obtain time series of historical traffic flow:
(2) using MHPSO to optimize the parameters of GRU neural network to get the optimal short-term traffic flow prediction model. The specific modeling steps are as follows:
  Step 1: initialize the parameters of particle swarm and GRU neural network. The parameters of particle swarm include population scale, population layers, iterations, learning factors, and the limited range of particle position and velocity. The initial values of particle position and velocity are random. The parameters of GRU neural network mainly include the number of neurons in each layer of neural network and the number of layers of hidden layer.
  Step 2: calculate the fitness value of each particle in the population and build the population hierarchy. The fitness function of particles in the population is defined as:
\begin{equation*} fit_{i} =\frac {1}{n}\sum {(Y_{i} -y_{i})^{2}},\quad i=1,2,3,\cdots,n\tag{11}\end{equation*}
    where $n$ is the population size, $Y_{i} $ is the sample output value, and $y_{i{\thinspace }}$ is the actual output value
  Step 3: calculate the fitness value of each particle
  Step 4: update the speed and position of particles according to formula (3) and (4)
  Step 5: if the end condition of iteration (good enough position or maximum number of iterations) is reached, it will end. Otherwise, turn to step 2 to continue iteration MHPSO-GRU network is to optimize the initial weight of deep GRU network through MHPSO, and then restore the optimal solution as the initial weight of deep GRU network. Because the optimal solution obtained by MHPSO is close to the final required weight and width of deep GRU network in the global scope, the deep GRU network trained by learning can have less learning time and improve its disadvantage of easily falling into local minimum, thus improving the performance of prediction using this model.

### B. Prediction Model of Aids Incidence Based on GRU Network

**FIGURE 5.**
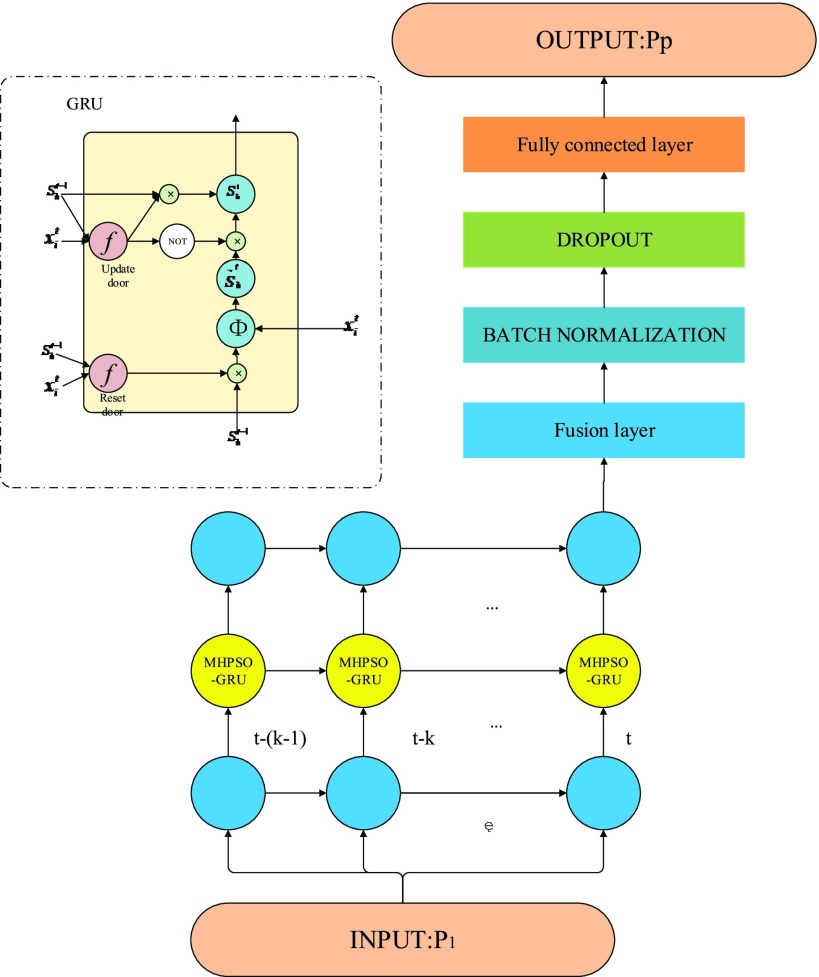
Prediction model of AIDS incidence based on MHPSO-GRU network.

This paper proposes a prediction model of HIV incidence based on GRU network. The network structure of the model is shown in Figure 5. Among them, the one month incidence of AIDS as a sample, recorded as $P$. $P$ is the incidence data of six months before the forecast month. The reason for setting the forecast interval to 6 months is based on the influence of date factors. As the input of RNN, $P$ needs to be divided into multiple subsequences, and the time step of segmentation takes the middle value.

## IV. MHPSO-GRU Network and Prediction of Aids Incidence

### A. GRU Neural Network Based on MHPSO Optimization

With the development of machine learning algorithm and data mining technology, the accuracy of incidence prediction is also increasing. In the face of complex and random systems, we often use one-dimensional time series data analysis, but this result is caused by many factors. It is necessary to study the influence of other factors in time series, which makes the pattern recognition of time series more important. In order to explore how these influencing factors affect the system and its internal relations, we can decompose the time series into some interpretable contents, such as trend item, cycle item, cycle component and irregular component, which can help us better simulate the time series. It is necessary to construct a topological space equivalent to the original system, that is, to project the influence factors hidden in the one-dimensional system that have not been observed into the geometric space, from the current state of the system, we can get the state of the next moment, that is, from the value at this time to the prediction value of the next moment of the time series. In this paper, GRU, a derivative network of RNN neural network, is used to predict the incidence of AIDS. The MHPSO-GRU network prediction model was constructed for the sequence of AIDS incidence.

### B. Prediction Model of Aids Based on MHPSO-GRU

**FIGURE 6.**
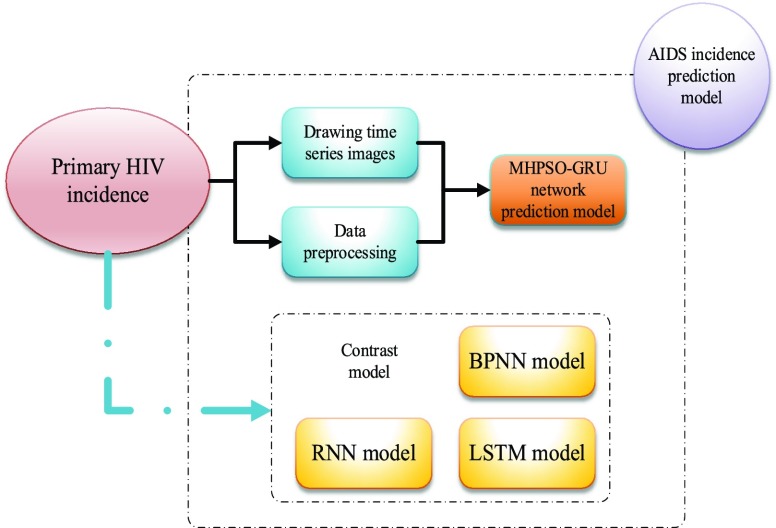
Prediction framework of AIDS incidence based on MHPSO-GRU model.

Cyclic neural network can not only predict the image of time series, but also process the length of any input or output. The parameters of MHPSO-GRU model are less than those of traditional RNN and LSTM model, so it is easier to program and put into use. The incidence of AIDS over the years is a time series, which is more suitable for RNN model training. In this paper, we build a prediction model of AIDS incidence based on MHPSO-GRU model, and the specific framework is shown in Figure 6.

In the prediction framework of AIDS incidence shown in Figure 6, this paper first collects the time series data of AIDS incidence in 2004-2011, draws the time series image, and analyzes the trend impact of AIDS incidence. In this paper, a model based on MHPSO-GRU neural network is established to predict the incidence of AIDS in the next few years. At the same time, we also use BPNN model, traditional RNN model and LSTM model to model and predict the future incidence of AIDS based on historical data, and analyze the differences and advantages and disadvantages of the four prediction methods.

## V. Model Performance Evaluation Experiment and Enalysis

### A. Experimental Data and Network Parameter Setting

The monthly cases of longitudinal AIDS collected from January 1, 2004 to April 30, 2018 in mainland China were collected from the National Notifiable Infectious Diseases Reporting System (2018). A total of 172 observations over 15 years were collated and summarized (table S1). Subsequently, in order to evaluate the maintainability and flexibility of each model developed, we used the data from January 1, 2004 to December 31, 2016 to train the basic parameters of the model, and used the remaining 16 data points to verify the model extrapolation ability. Our current research is not necessarily ethically approved or agreed, because the data do not have personally identifiable information and are publicly available.

### B. Test Results and Comparison

**FIGURE 7.**
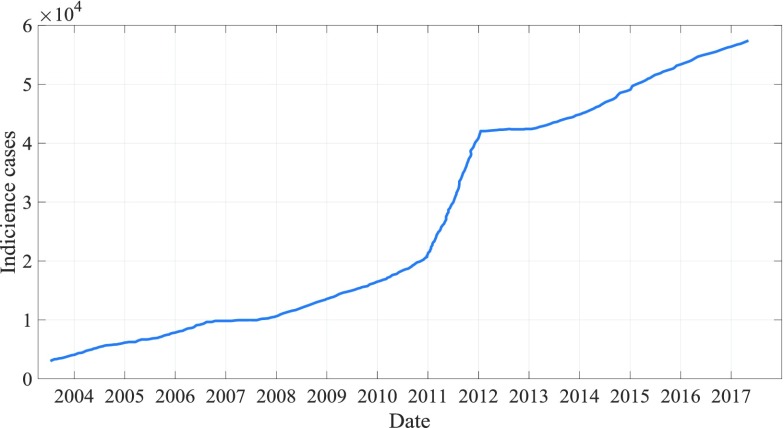
Time series of monthly AIDS cases from 2004 to 2017.

Between January 2004 and April 2018, there were 458078 AIDS cases in total, with an average of 2664 cases per month, 143 standard errors, and an average annual incidence of 2 per 100000 people. In 2017, China reported 57194 HIV cases per month, 18 times more than in 2004 (only 3054 cases). In addition, when HP filter is used to remove the short-term monthly impact of AIDS incidence to analyze long-term trend and periodicity, a trough is usually observed in January and February of each year, while the case is frequently reported in December of each year. In the remaining months, the number of reported cases remained stable and the volatility has become more pronounced since 2010 (Figure 7). In addition, in the past 14 years, the number of reported AIDS cases has increased significantly, with an annual increase of about 14.384%.

Figure 7 is a time series image of the monthly AIDS medical records from 2004 to 2017. From the figure, we can see that the incidence of cases is gradually increasing. The rate of increase from 2004 to 2010 is relatively slow, and the rate of increase from 2011 to 2012 is suddenly increasing, but the trend of increase from 2013 to 2017 is generally consistent with that from 2004 to 2010.

From January 2004 to April 2018, there were a total of 458,078 AIDS cases, with an average of 2664 cases per month and a standard error of 143 cases. The average annual incidence rate was 2 per 100,000 people2. In 2017, the number of AIDS cases reported each month in mainland China reached 57,194, which was more than 18 times the number in 2004 (only 3054 cases). In addition, when HP filtering is used to remove the short-term monthly effects of AIDS incidence to analyze long-term trends and periodicity, a trough is usually observed in January and February each year, and the case is frequently reported in December each year. The number of notified cases remained stable for the remaining months, and this fluctuation has become more pronounced since 2010. In addition, the number of reported AIDS cases has shown a significant upward trend over the past 14 years, and has increased by approximately 14.384% per year.

The above figure is a time series image of the incidence of AIDS from January 2004 to January 2018. As shown in the figure, the incidence of AIDS shows a gradual upward trend, which seems to be more obvious since 2010, with a downward trend in 2018. In addition, periodic fluctuations also occur in the same period.

For the prediction experiment of AIDS incidence based on MHPSO-GRU network, the model parameter settings are shown in Table 2.

**TABLE 1:** Predicted Network Structure

Network layer	Cell number
Input $P_{1} $	(1,6)
GRU	300
Fusion layer	400
BATCH NORMALOZATON	(1,6)
DROPOUT	400
Fully connected layer	100
Output: $P_{p} $	6

**TABLE 2:** Design Values of Predicted Network Parameters

Parameter	Parameter value
Input step number	6
Input step size	16
Number of samples per batch	30
optimization method	RMSprop
Learning rate	0.001
Dropout probability	0.6

For the actual change curve and prediction curve of AIDS incidence from January 2017 to April 2018, see Figure 9. MAPE is predicted to be 8.15%. The actual and predicted curves of AIDS incidence from January 2009 to January 2018 are shown in Figure 9, of which the predicted MAPE is 8.21%. From the comparison of the results in Figure 9 and Figure 11, it can be seen that the difference between the whole prediction curve and the actual curve is not big, and the whole prediction error is quite stable within one or ten years, without large deviation, which proves that the method in this paper is effective.

**FIGURE 8.**
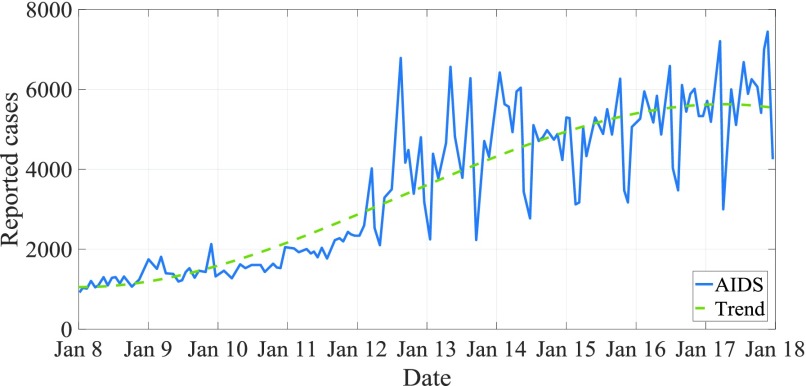
Sequence of monthly HIV incidence reported and filtered using HIV filtering method from January 2004 to January 2018.

**FIGURE 9.**
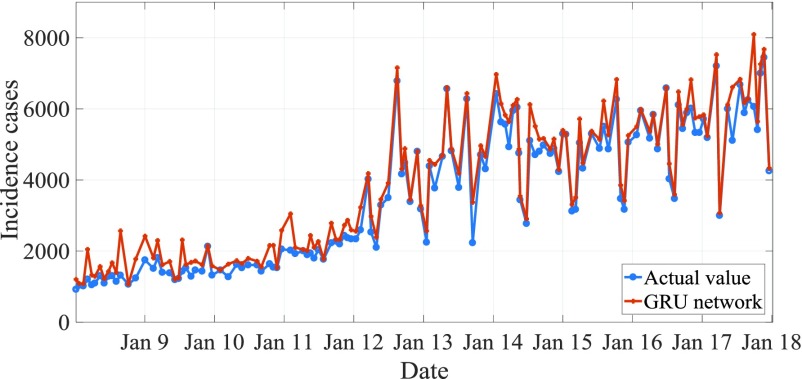
Actual and predicted curve of AIDS incidence from January 2009 to January 2018.

**FIGURE 10.**
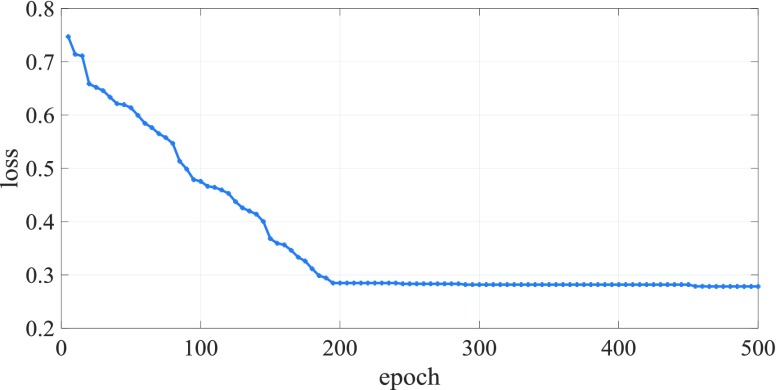
Training loss function iteration diagram of AIDS incidence prediction model.

**FIGURE 11.**
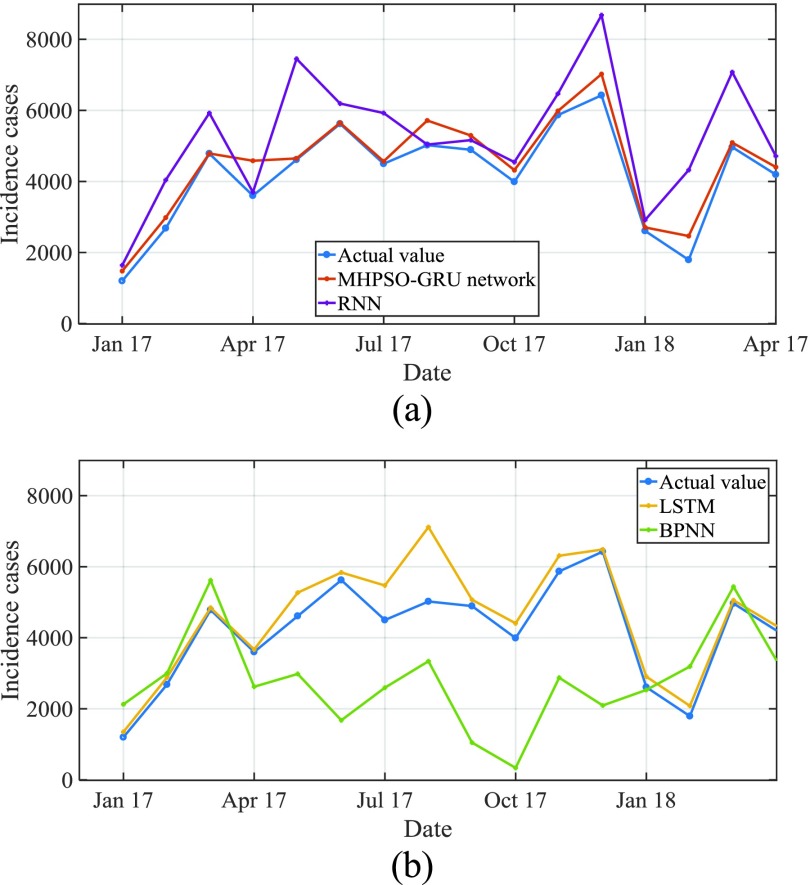
Comparison of estimated incidence between four selected models and observations from January 2017 to April 2018.

Figure 10 shows the image of the training loss function of the AIDS incidence prediction model changing with the number of iterations. From the above figure, it can be seen that the MHPSO-GRU based AIDS incidence prediction model tends to be stable when the number of training reaches 400, and then the loss function value is close to 0.28, indicating that the prediction effect is better.

### C. Performance Comparison

**FIGURE 12.**
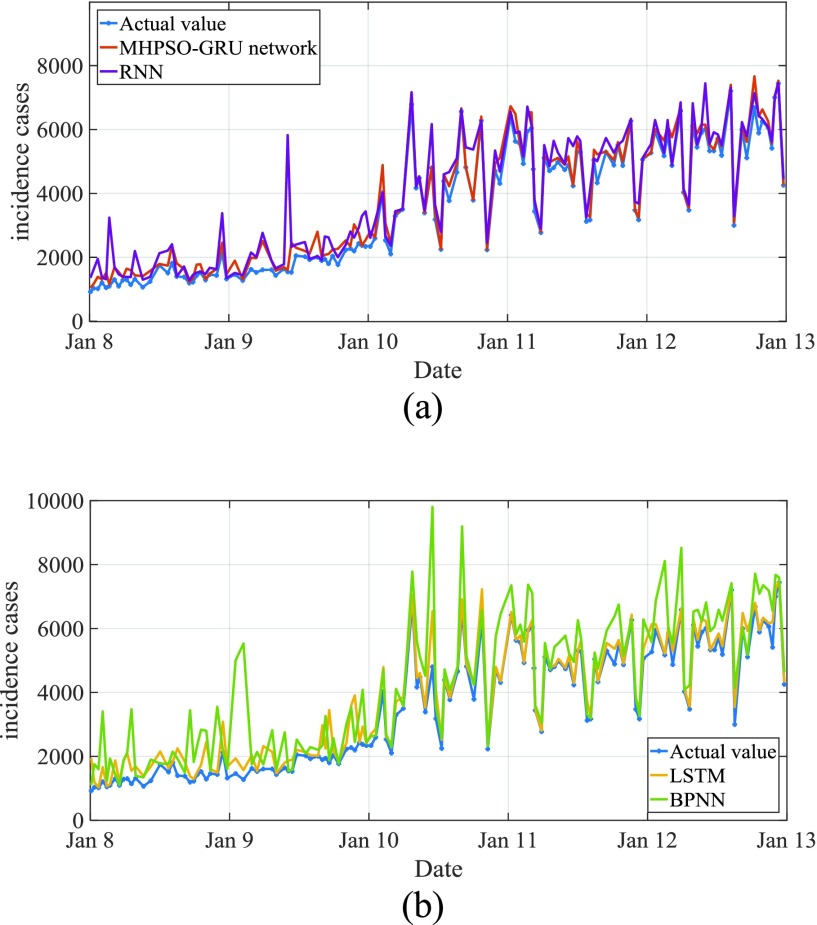
Comparison of simulated and estimated incidence cases between selected four models and observations.

Table 3 shows the prediction ability and efficiency of the four best fit models selected. We found that compared with MAE, MAPE, mer and RMSE, the fitting results of basic BPNN model were the lowest, so as to analyze the future epidemic mode of known cases of AIDS. In the out of sample prediction, AIDS had the maximum value of the above four indicators. The MHPSO-GRU model is considered to be the most suitable model for prediction in the derivative model based on the full consideration of the performance simulated and predicted by these models (Figure 11 and Figure 12). Subsequently, this hybrid approach was re established and data from the whole report were used to simulate the time behavior of AIDS in the near future. As can be seen from table 7, it shows that the disease seems to be on the rise from May 2018 to December 2020.

**TABLE 3:** Comparison of Fitting Performance in Samples of the Model

Models	Fitting performance				Predicted performance			
	MAE	MAPE	MER	RMSE	MAE	MAPE	MER	RMSE
MHPSO-GRU	50.671	0.037	0.035	148.089	278.875	0.167	0.076	771.710
LSTM	54.884	0.053	0.022	142.481	1628.679	0.337	0.350	2019.439
RNN	149.388	0.132	0.059	224.292	539.027	0.144	0.116	799.314
BPNN	335.568	0.193	0.126	502.502	1013.006	0.290	0.218	1301.985

MHPSO-GRU, MHPSO-Gated Recurrent Unit; LSRM, Long Short-Term Memory; RNN, Recurrent neural network; BPNN, Back Propagation Neural Network; MAE, Mean absolute error; MAPE, mean absolute percentage error; mer, mean error rate; RMSE, root mean square error.9

Figure 11 shows the predicted incidence of AIDS by MHPSO-GRU model, LSTM model, RNN model and BPNN model. It can be seen that BPNN model has the worst prediction effect and the largest deviation from the actual data. The prediction error of RNN model is large, that of LSTM model is small, and that of MHPSO-GRU model is minimum.

Figure 12 shows the global prediction of AIDS incidence by four algorithms: BPNN model, RNN model, LSTM model and MHPSO-GRU model. The blue line is the time sequence of actual incidence of AIDS, the red line is the prediction sequence of MHPSO-GRU model, the yellow line is the prediction sequence image of LSTM model, the purple line is the prediction sequence of RNN model, and the green line is the prediction sequence of BPNN model In addition to BPNN model, other models have good prediction effect, but MHPSO-GRU model has better prediction effect. As described above, the curve (solid red line) fitted by the MHPSO-GRU method can better approximate the reality (solid blue line) compared to other curves.

**FIGURE 13.**
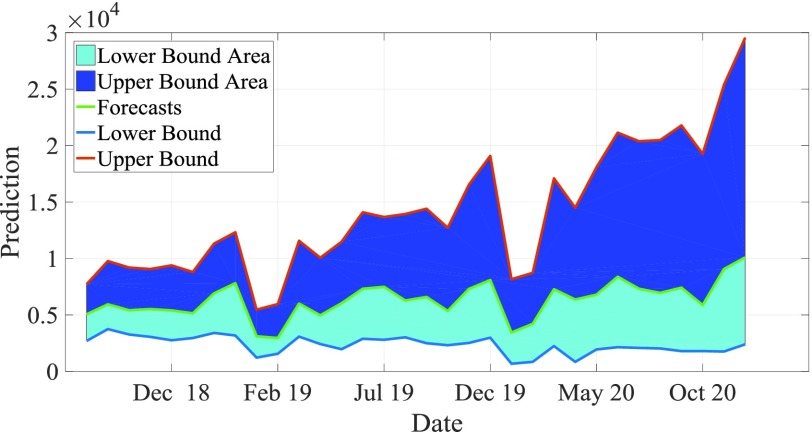
Prediction value and prediction interval of MHPSO-GRU model.

Table 4 shows the prediction value and 95% prediction interval of 2018–2020 based on MHPSO-GRU model, and figure 13 shows the prediction value and prediction interval of 2018-2020. From the comprehensive table and figure, we can see that the incidence of AIDS in the future is still rising, and the situation of AIDS prevention and control is still very serious.

**TABLE 4:** Forecasts With the Best-Performing ARIMA-NAR Hybrid Techninque Between May 2018 and December 2020

Date	Forecasts	95% confidence limits	Date	Forecasts	95% confidence limits
May-18	5057	[2698, 7719]	Sep-19	6591	[2496, 14407]
Jun-18	5943	[3748, 9768]	Oct-19	5355	[2319, 12723]
Jul-18	5418	[3270, 9197]	Nov-19	7311	[2522, 16548]
Aug-18	5521	[3058, 9067]	Dec-19	8092	[2987, 19082]
Sep-18	5401	[2758, 9391]	Jan-20	3445	[680, 8161]
Oct-18	5147	[2955, 8802]	Feb-20	4214	[846, 8730]
Nov-18	6913	[3408, 11317]	Mar-20	7277	[2244, 17095]
Dec-18	7813	[3182, 12322]	Apr-20	6366	[842, 14496]
Jan-19	3115	[1221, 5468]	May-20	6794	[1944, 18073]
Feb-19	2956	[1563, 5963]	Jun-20	8384	[2145, 21143]
Mar-19	6008	[3081, 11567]	Jul-20	7322	[2081, 20386]
Apr-19	4961	[2423, 10065]	Aug-20	6954	[2031, 20488]
May-19	6038	[1966, 11476]	Sep-20	7417	[1795, 21785]
Jun-19	7320	[2890, 14091]	Oct-20	5870	[1801, 19269]
Jul-19	7493	[2802, 13673]	Nov-20	9064	[1759, 25371]
Aug-19	6275	[3018, 13938]	Dec-20	10099	[2398, 29549]

To sum up, according to the longitudinal data of known cases of AIDS reported, we use four useful methods, BPNN, RNN, LSTM, MHPSO-GRU, to model and solve. Figure 12 is a comparison chart of the simulated and estimated incidence rate between the selected four models and observations. The red line is the prediction sequence of the MHPSO-GRU model, and the blue line is the actual incidence rate curve. It is seen that the error of the red line and the blue line is smaller. Table 3 shows the prediction performance comparison of sample fitting of the four models. Mae of MHPSO-GRU network model is 278.875, MAPE is. 0.167, Mer is 0.076, RMSE is 771.710, which is the best of the four effective methods. This may be a useful technology for estimating the future behavior of AIDS.

## VI. Conclusion

In the past decades, the incidence rate and mortality rate of AIDS has been increasing. The situation of countries and regions in the world has worsened. The premise of effective containment and intervention strategies for AIDS by relevant organizations is accurate identification and early warning of the future long-term track of this disease behavior.

The advantages of our research include the amazing findings gathered from the AIDS incidence rate data collected from January 2004 to April 2018. The validation of these data has been supported by China’s mandatory infectious disease surveillance system. It can be seen that figure 8 shows that the incidence rate of AIDS in China is increasing. This work can study the future trend of AIDS incidence rate in China, and provide corresponding treatment strategies and intervention measures. In the future work, we plan to study the trend of AIDS rate in other areas to prove that our model is effective and universal.

Even so, there are some limitations in this work. First, the data cover the period including policy interventions (2010). Although the results of the first choice model are satisfactory, the actual impact of the model’s accuracy before and after the sudden rise in incidence rate of AIDS is still unknown. Secondly, in the construction of RNN model, due to the lack of mature theoretical guidance on the optimal number of hidden cells, feedback delay and other key parameters. Although mhpso algorithm is used to improve the number of singular numbers in this paper, its specific simulation and prediction process is largely unknown. Third, it is estimated that 32.4% of AIDS patients in China do not know their infection status. Therefore, the actual situation may be more serious than the estimated situation. Fourthly, many complex factors, including unpredictable factors, may lead to the occurrence and spread of AIDS. The prediction model we built only relies on the known AIDS cases in the past, without considering other factors to predict the unknown cases. Therefore, if conditions permit, factors related to the occurrence and development of AIDS are expected to be used together to assess the epidemic. Finally, the prediction model is based on the cases in China from January 2004 to April 2018. Therefore, the analysis of the epidemic only represents the situation and trend of AIDS in the country. After being recorded, this method has great benefits for other regions and infectious diseases.

To sum up, we use BPNN, RNN, LSTM respectively according to the longitudinal data of known AIDS cases reported, Four useful methods of MHPSO-GRU are used to model and solve the problem. It is found that the performance of MHPSO-GRU network model is the best. This may be a useful technique to estimate the temporary behavior of AIDS, so it can help health policy makers to reasonably allocate health resources The prevention and control plan of the disease shall be properly formulated. In addition, the number of new HIV infections will continue to increase and is already high, and these issues deserve urgent strategic attention in the context of properly adopted policies.
